# First Multi-Center All-Comers Study for the Aquablation Procedure

**DOI:** 10.3390/jcm9020603

**Published:** 2020-02-24

**Authors:** Thorsten Bach, Peter Gilling, Albert El Hajj, Paul Anderson, Neil Barber

**Affiliations:** 1 Asklepios Westklinikum Hamburg-Rissen, 22559 Hamburg, Germany; 2 Tauranga Urology Research Ltd, 3112 Tauranga, New Zealand; gilling.peter@gmail.com; 3 American University of Beirut Medical Center, 1107 2020 Beirut, Lebanon; ae67@aub.edu.lb; 4 Royal Melbourne Hospital, Melbourne, Parkville, VIC 3050, Australia; mr.paul.anderson@gmail.com; 5 Frimley Park Hospital, Trust Lead for Urology, Frimley, Camberley GU16 7UJ, UK; neil.barber@nhs.net

**Keywords:** robotic surgery, benign prostatic hyperplasia, lower urinary tract symptoms

## Abstract

Waterjet-based prostate resection (Aquablation procedure) is an increasingly recognized treatment for symptomatic benign prostatic hyperplasia (BPH). We confirmed the safety and effectiveness of the Aquablation procedure in the commercial setting in 178 men at five sites. The mean prostate volume was 59 cc. The procedure time averaged 24 min and total anesthesia duration was 50 min. The International Prostate Symptom Score (IPSS) decreased from 21.6 at the baseline to 6.5 at the 12-month follow-up, a 15.3-point improvement (*p* < 0.0001). The maximum urinary flow rate increased from 10 cc/s at the baseline to 20.8 cc/s at month 12 (increase of 11.8 cc, *p* < 0.0001). Ejaculatory function was relatively preserved. Prostate volume assessed with transrectal ultrasound decreased 36% by month three. Five patients (2.7%) underwent a transfusion in the first week after the procedure. Real-world evidence shows that Aquablation is safe and effective for the treatment of BPH.

## 1. Introduction

Surgical treatment approaches for men with moderate-to-severe benign prostatic hyperplasia (BPH) include resection, enucleation or energy-based tissue modification (so-called “ablation”). The choice of technique is largely determined by prostate volume. For men with very large prostates (>80 cc), guidelines suggest that transurethral resection (TURP) should be avoided. Procedures such as a simple open or laparoscopic/robotic prostatectomy (SP) [1,2,3], Holmium laser enucleation of the prostate (HoLEP) [4], or photoselective vaporization (PVP) [5] are more commonly performed. However, these procedures are associated with substantial morbidity and have a relatively steep learning curve. The downside of SP is a longer hospital stay, a high risk of bleeding (including transfusions) and a longer duration of catheterization. HoLEP is challenging to learn [6,7] and is therefore available at few centers. PVP for large prostates is expensive and has long operative times and a relatively high re-operation rate with larger glands [8].

The Aquablation procedure is a recently available technique that integrates real-time ultrasound imaging with a robotically executed, surgeon-guided, high-velocity waterjet to precisely resect prostate tissue. Initial studies have demonstrated efficacy similar to TURP, with a much lower risk of sexual side effects [9,10,11]. Herein, we report real-world safety and effectiveness from the use of Aquablation in a standard commercial setting.

## 2. Materials and Methods

### 2.1. Trial Design and Participants

OPEN WATER (NCT02974751) is a prospective, multicenter, single-arm, open-label, international clinical trial of the Aquablation procedure for the treatment of lower urinary tract symptoms (LUTS) due to BPH. To be included, men had to have a diagnosis of LUTS due to BPH and a prostate size between 20 and 150 cc. Men were excluded if they were unable to stop anticoagulants and antiplatelet agents perioperatively or had a bleeding disorder, had a history of gross hematuria, were using systemic immune suppressants, had a contraindication to both general and spinal anesthesia, were unwilling to accept transfusion if required, or had any severe illness that could prevent complete follow-up. Patients with prior BPH surgery were not excluded.

All study sites obtained Institutional Review Board/Ethics Committee approval prior to study-related consent. The study was sponsored by the device manufacturer (PROCEPT BioRobotics, Redwood City, CA, USA). The sponsor had no say in the selection of trial subjects. The physician participants were trained in use of the study device prior to study start. Aquablation procedure experience entering the study with the participating surgeons was PG-33, PA-25, NB-22, AH-7, and TB-0.

At the baseline and at the follow-up (3 and 12 months), subjects completed the following: IPSS, Incontinence Severity Index, Pain Intensity Scale, Quality of Recovery Visual Analog Scale, International Index of Erectile Function (IIEF-15 [12]), the Male Sexual Health Questionnaire (MSHQ-EjD [13]), uroflowmetry, and post-void residual (PVR) volume measurements.

The Aquablation procedure was performed as described previously [9]. Briefly, after the induction of general or spinal anesthesia, a 24F handpiece was inserted into the prostatic urethra and secured into place using a bed-mounted rigid arm. Under real-time transrectal ultrasound (TRUS) guidance, the surgeon defined the target anatomic resection contour on a computer console. The tissue was then ablated robotically utilizing a high-velocity waterjet that moves in a controlled manner from the bladder to the verumontanum. The contour of the tissue to be ablated was drawn to safeguard the bladder neck, ejaculatory ducts, and urinary sphincter. Based on the surgeon’s assessment of tissue resection, additional passes were planned and executed during the same session. The most common (90%) post-operative hemostasis technique was robust catheter traction without electrocautery.

### 2.2. Data and Study Monitoring

All study data were collected using study-specific case report forms. Remote data monitoring (i.e., identification of potential data errors) was performed by the study sponsor. Adverse events deemed potentially related to the procedure or device were collected, including start/stop date and relatedness to the procedure or device. The site investigator also assigned a Clavien–Dindo grade [14] for each adverse event.

### 2.3. Study Endpoints and Statistical Analysis

The study’s primary efficacy endpoint was the change in total IPSS score from baseline to 3 months. The secondary endpoints included the following: (1) Proportion of subjects who were sexually active at the baseline and experienced either ejaculatory or erectile dysfunction at 3 months, change from the baseline to 3 months in maximal flow rate (Qmax), prostate specific antigen (PSA) level, post-void residual (PVR), total MSHQ score and selected IIEF-5 score. The degree of dysuria was collected on a 0 (not at all) to 5 (almost always) scale.

Safety analysis focused on all patients undergoing the Aquablation procedure. Effectiveness analysis focused on patients with a 3-month follow-up. Changes in continuous measures were evaluated using a t test. Changes in binary measures were evaluated using paired or unpaired chi-squared or Fisher’s tests. All statistical analysis was performed using R [15].

### 2.4. Data Availability

De-identified raw study data are available for sharing for valid scientific requests.

### 2.5. Research Ethics

Sites participating in this interventional study obtained ethics committee approval prior to first enrollment. Ethics committees are listed in Appendix A.

## 3. Results

A total of 178 men were enrolled at five centers (one each in the United Kingdom, Germany, Australia, New Zealand, and Lebanon) between September 2017 and December 2018. All patients were eligible for the study, except for one subject, who had a coagulopathy that was undiagnosed at the time of the procedure. Of the 178 subjects who enrolled and underwent the study procedure, by month 12, 30 subjects were lost to follow-up, three voluntarily withdrew and one died of non-urologic cause. Loss to follow-up at one site was high due to political instability.

Baseline patient characteristics are summarized in Table 1. The mean age was 68 years and baseline IPSS was 21.7. The mean prostate volume, as estimated by TRUS, was 59 cc (range 20–148 cc). Meanwhile, 126 (71%) were sexually active. A middle lobe was present in 59% of cases; of these 72% had an intravesical component.

The mean operative duration (handpiece placement to urinary catheter placement) was 24 min (range 8–70 min). The mean total anesthesia duration was 50 min (range 22–115 min). Operative time and anesthesia duration increased by 0.13 and 0.2 min per cubic centimeter of prostate tissue (*p* = 0.0002 and *p* < 0.0001), respectively. Using linear regression, the estimated mean operative times for prostates of sizes 50, 100 and 150 cc were 23, 29 and 36 min, respectively.

Post-procedure hemostasis was achieved utilizing a urinary catheter with the surgeon’s preference of bladder neck traction. In 19 (10.7%) cases, hemostasis was augmented with focal cautery. The median catheterization time following surgery was 1.9 days. Hospital length of stay averaged 2.2 days (range 0–12). At non-German sites, 81% had a hospital length of stay of 2 days or less. Hemoglobin levels decreased from a mean of 14.7 at baseline to 12.8 prior to discharge (drop of 2 g/dL, *p* < 0.0001), Table 2. Five patients (2.7%) underwent transfusion in the first week after the procedure; of these, one was for delayed (day 6) bleeding. Fourteen (7.9%) patients were taken back to the operating room for post-procedure bleeding; hemostasis was achieved with cautery at the bladder neck or prostatic fossa. One patient returned twice to the operating room (OR) for clot evacuation.

**Figure 1:**
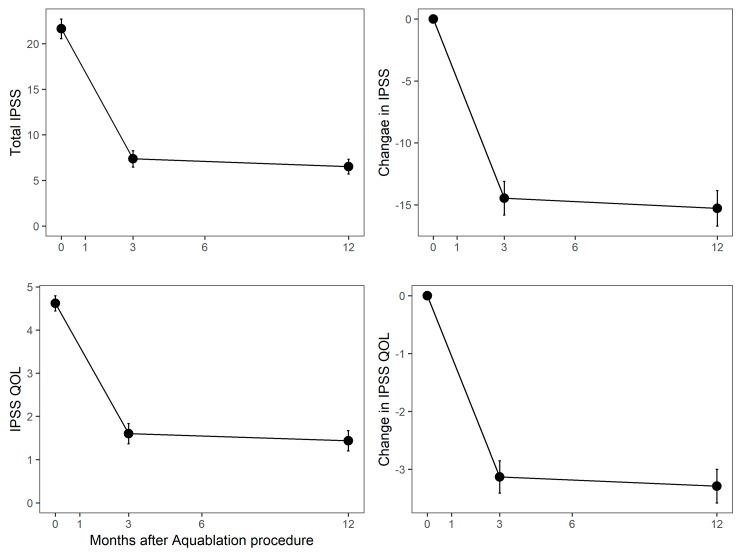
Absolute (y-axis) and change (y-axis) in International Prostate Symptom Score (IPSS) and IPSS QOL. Population means are shown at the left and score changes at the right. Months after Aquablation procedure is the y-axis in all graphs.

Mean (SD) IPSS improved from 21.7 (7.1) at the baseline to 7.1 (5.8) at the 3-month follow-up (a 14.5-point improvement, *p* < 0.0001), and 6.4 (4.8) at the 12-month follow-up (a 15.3-point improvement, *p* < 0.0001), Figure 1. Mean (SD) IPSS QOL scores improved from 4.7 (1.1) at the baseline to 1.5 (1.4) at the 3-month follow-up, a 3.1-point improvement (*p* < 0.0001), and 1.4 (1.4) at the 12-month follow-up (a 3.3-point improvement, *p* < 0.0001). IPSS storage and voiding scales also improved significantly (*p* < 0.0001) at 3 and 12 months. The 3-month and 12-month IPSS scores were independent of baseline IPSS.

Baseline IPSS scores were unavailable in nine men; of these, seven were using a urinary catheter at the baseline and two cases had incomplete questionnaire responses. Of the seven on catheter at the baseline, mean IPSS at the 12-month follow-up was 10.

**Figure 2:**
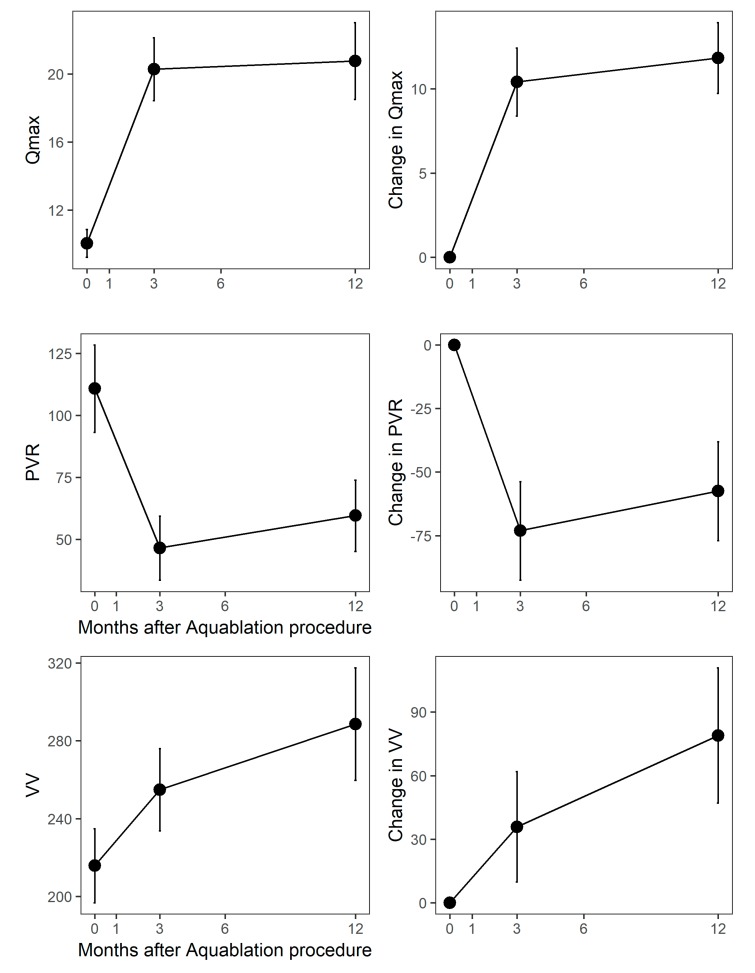
Absolute (y-axis) and change (y-axis) in uroflow parameters after Aquablation. Qmax = maximal urinary flow rate; PVR = post-void residual; VV = voiding volume. Population means are shown at the left; change from the baseline is shown at the right. Months after Aquablation procedure is the y-axis in all graphs.

Maximum urinary flow rate increased from 9.9 (5.3) cc/sec at baseline to 20.3 (11.4) cc/sec at month 3 and 20.8 (11.2) cc/s at month 12 (both increases *p* < 0.0001 vs. the baseline), Figure 2. Post-void residual improved from 108 (108) to 47 (77) cc at three months and 61 (74) cc at 12 months (both *p* < 0.0001 vs. the baseline).

**Figure 3:**
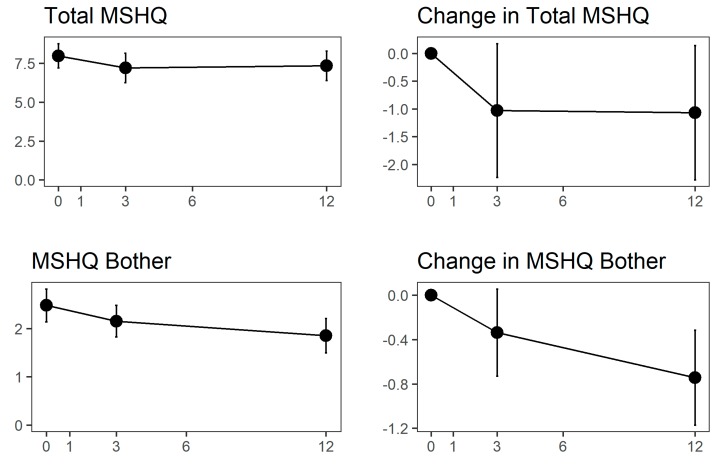
Absolute (y-axis) and change (y-axis) in ejaculatory function as measured by Men’s Sexual Health Questionnaire (MSHQ-EjD) scores. Population means are shown at the left; changes from baseline are shown at the right. Months after Aquablation procedure is the y-axis in all graphs.

**Figure 4:**
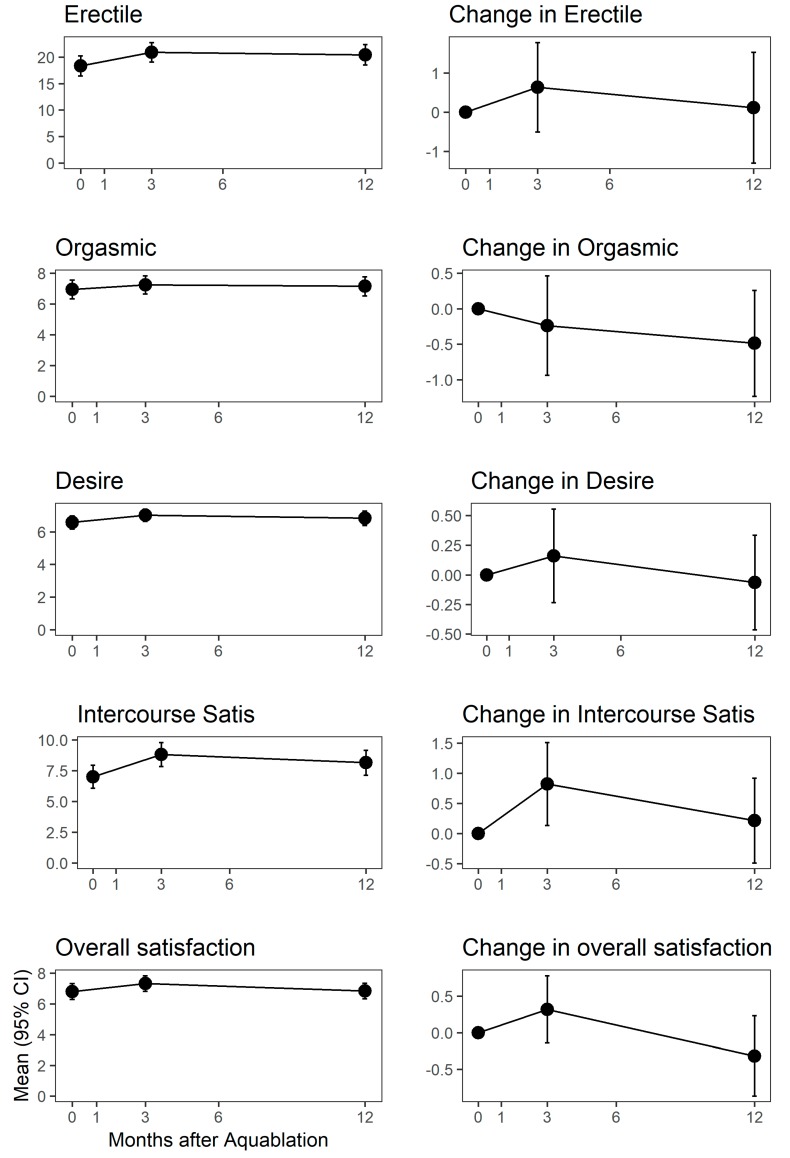
Absolute (y-axis) and change (y-axis) in sexual function scores from the baseline to month 12. Top two plots are MSHQ-Ejd score and bother score. Bottom five plots are IIEF-15 subdomains. Months after Aquablation procedure is the y-axis in all graphs.

Amongst 92 men who were sexually active at both the baseline and the 12-month follow-up study visit, MSHQ-EjD score changed by −1 point at 3 months (*p* = 0.0994) and by −1.1 points at 12 months (*p* = 0.0884), Figure 3. MSHQ bother changed by −0.3 and −0.7 points at 3 and 12 months (*p* = 0.0962 and 0.0025). IIEF-15 subdomain scores remained stable throughout month 3, Figure 4.

141 men had a transrectal ultrasound examination at both the baseline and after 3 months. Mean (SD) prostate size measured was 59 cc (27) at the baseline and 35 cc (20) at the follow-up, representing a 36% decrease in volume (*p* < 0.0001). For subjects with a baseline PSA ≥2, the 3-month drop in PSA was 41.5%.

In men with a 12-month follow-up, leakage of urine was reported by 68% at the baseline and 55% at the 12-month follow-up. Incontinence (ISI) scores amongst those reporting leakage improved non-significantly from 3.3 at the baseline to 2.5 at the 12-month follow-up (change of −0.7 points, *p* = 0.0366). Dysuria of any frequency was reported by 51% at the baseline and 29% at 3-month follow-up (*p* < 0.0001, McNemar test). For men reporting dysuria, mean pain levels were 3.5 at the baseline and 2.4 at 3-month follow-up (1–10 scale). Pelvic pain levels, assessed preoperatively and at 3 months postoperatively using a 0–10 numeric rating scale, averaged 1.3 and 0.4 respectively.

Eighty-two patients (46%) were taking medications for BPH preoperatively. By month 3, all but eight had stopped these medications. Five patients began alpha blockers during the follow-up. Nineteen were taking 5-alpha reductase inhibitors at the baseline; of these, all but one stopped and one started ARIs during follow-up.

Sixty-nine adverse events were reported in 56 subjects. Of these there were 33 grade 1 events, 15 grade 2 events, five grade 3a events and 16 grade 3b events (Table 3). As noted above, five of 182 (2.7%) patients underwent transfusion in the week following Aquablation; 14 (7.9%) patients were taken back to the OR for postoperative hemostasis management. In one case, the subject had a preoperative coagulopathy; postoperatively, the subject was diagnosed with a fibrin dysfunction using thromboelastometry. Additionally, one patient had rectal perforation requiring a temporary colostomy and 3 patients had meatal stenosis or stricture requiring a procedure. Fifteen patients (8%) reported ejaculatory dysfunction (including one case of climacturia) and one (1%) reported erectile dysfunction with a one-point drop in total Sexual Health Inventory for Men (SHIM) score. No patient underwent TURP or other procedures for BPH symptoms.

## 4. Discussion

This is the first multi-center publication of outcomes after the Aquablation procedure performed in a commercial, i.e., non-clinical trial-related, setting. Our results indicate that the Aquablation procedure is safe and effective for men with LUTS due to BPH, and that the results obtained in the commercial setting replicate those observed in previously reported trials of this procedure [11,16]. Specifically, men undergoing Aquablation treatment in our study were of similar age and had a similar prostate size compared to prior trials, but had a higher incidence of leakage and a history of urinary retention compared to the landmark WATER randomized trial. This is likely due to the lack of prostate size restriction in our study compared with WATER, which restricted prostate size to a 30-80 cc range. The baseline symptom scores were similar to WATER and indicated a typically high rate of symptoms and dissatisfaction with urinary function. Seven of 10 men are sexually active at the baseline. 

The surgical parameters were typical for the Aquablation procedure, with a mean anesthesia time of 50 min and operative time of 24 min. For a 100 cc prostate, the mean operative time was 29 min, which is substantially lower than that observed for open prostatectomy (95 min [17]), HoLEP (91 min [18]), or PVP (93 min [19]) in similarly sized prostates. The length of stay in hospital was <2 days in most (81%) cases.

We observed high levels of symptom relief consistent with prior Aquablation trials and, more generally, with results observed from most resective techniques. Large improvements were seen with all other efficacy measures, including IPSS QOL, IPSS subscores, maximum urinary flow rate, post-void residual and symptoms related to urinary leakage. Improvement in IPSS was independent of baseline score, indicating “ceiling effects” (i.e., near maximal improvement) for symptom reduction. Most patients were able to stop medications related to BPH. Postoperative pain levels were very low, and dysuria related to BPH decreased.

Previous reports of Aquablation procedure outcomes have included a much lower rate of negative impact on sexual function, and our observations are similar. Men undergoing resective procedures of the prostate face an approximate 2/3 chance of post-procedure anejaculation. Consistent with prior Aquablation studies, we observed a far lower rate (8%) of anejaculation and minimal overall impact on MSHQ-EjD scores. The low rate is likely related to precise image-based and robotically executed targeting of tissue resection avoiding damage to the ejaculatory ducts.

The most common adverse event observed was post-procedure bleeding; five of 182 (2.7%) patients underwent transfusion in the week following Aquablation; 14 (7.9%) patients were taken back to the OR for postoperative hemostasis management, typically involving cautery. Given the large mean prostate size in our study (60 cc), which predisposes to bleeding, this rate seems acceptable. Since enrollment was completed, changes in approach to hemostasis after Aquablation have included modest post-Aquablation non-resective cautery at the bladder neck. This appears to have reduced the intensity of bleeding after Aquablation, but supportive data are not yet available. It is important to note that the surgical approach without electrocautery was used in 90% of the patients involved in the study.

Advantages of our study include its prospective multicenter design and recruitment of patients in a non-clinical trial setting. The study involved surgeons with both high and low levels of experience with the Aquablation procedure. Similar levels of symptom relief were seen independent of surgical experience (not shown). The disadvantages of our study include a lack of a concurrent control group and a relatively short-term efficacy follow-up. In light of very positive outcomes from the WATER randomized trial comparing Aquablation and TURP procedures [11], a control group is not necessary.

## 5. Conclusions

Real-world evidence shows that the Aquablation procedure is safe and effective for the treatment of symptomatic benign prostatic hyperplasia. 

## Figures and Tables

**Table 1:** Baseline characteristics (*n* = 178).

Characteristic	Statistic *
Age, years, mean (SD, range)	67.7 (8.5, 38–88)
Leakage of urine, *n* (%)	122 (68.5%)
History of urinary retention	36 (20.2%)
History of prostate cancer	1 (0.6%)
Bladder outlet obstruction	154 (86.5%)
Prostate size (TRUS, cc)	59.3 (26.9, 20–148)
Middle lobe	106 (59.6%)
Intravesical component	77/106 (72.6%)
Baseline questionnaires **	
IPSS score, (mean, SD)	21.6 (7.2, 0–35)
IPSS QOL, (mean, SD)	4.6 (1.2, 1–6)
Sexually active, *n* (%) [MSHQ-EjD]	126 (70.8%)
MSHQ-EjD (mean, SD) ***	8 (3.9, 1–15)
Laboratory	
Baseline hemoglobin	14.7 (1.3, 10–18)
Prostate specific antigen, g/dL; (mean, SD)	4.3 (3.9, 0.1–23)

* Continuous values shown as mean (SD, range); ** Questionnaire scores reported only in those with 3-month follow-up data (*n* = 161); *** Sexually active men only.

**Table 2:** Operative characteristics.

Characteristic	Statistic *
Intraoperative, min	
Anesthesia duration	50.5 (19.8, 22–115)
Operative time	24.2 (11.3, 8–70)
Handpiece in/out time	18.9 (8.5, 7–60)
Postoperative	
Discharge hemoglobin	12.8 (1.8, 7.2–17)
Change in hemoglobin	−2 (1.7, −7.5–0.5)
Length of stay, mean (SD, range)	2.2 (1.7, 0–12)

* Values shown as mean (SD, range).

**Table 3:** Distribution of perioperative complications categorized by Clavien–Dindo grade.

CD Grade	Term	Events	Subjects	Rate
1	Bleeding	3	3	2%
	Cardiovascular	4	4	2%
	Ejaculatory dysfunction	14	14	8%
	Erectile dysfunction	1	1	1%
	Infection	2	2	1%
	Other	1	1	1%
	Paraphimosis	1	1	1%
	Urinary incontinence	1	1	1%
	Urinary retention	6	6	3%
2	Bleeding	3	3	2%
	Cardiovascular	1	1	1%
	Infection	8	7	4%
	Other	1	1	1%
	Urinary retention	2	2	1%
3a	Ejaculatory dysfunction (climacturia *)	1	1	1%
	Infection	1	1	1%
	Meatal stenosis	2	2	1%
	Poor urinary flow	1	1	1%
3b	Bleeding	14	13	7%
	Rectal perforation	1	1	1%
	Urethral stricture	1	1	1%

* Underwent cystoscopy, no cause discovered.

## Appendix A

**Table A1:** Ethics committees overseeing this study are listed below.

Site No.	Institution	Primary Investigator	EC Information
01	Frimley Park Hospital	Mr. Neil Barber	NHS Health Research Authority (REC)
02	Asklepios Klinikum Harburg	Prof. Dr. med. Thorsten Bach	Ethics Committee of the Hamburg Medical Association
04	Tauranga Hospital	Prof. Peter Gilling	Health and Disability Ethics Committees Ministry of Health (Northern B)
05	American University of Beirut Medical Center	Dr. Albert El-Hajj	American University of Beirut Institutional Review Board
06	Royal Melbourne Hospital	Mr. Paul Anderson	Melbourne Health Human Research Ethics Committee
